# Supplementary material for: Diversity of Alternaria Section Nimbya in Iran, with the Description of Eight New Species
Source: J Fungi (Basel). 2025 Mar 15;11(3):225. doi: 10.3390/jof11030225 (PMC11943149; doi:10.3390/jof11030225)
Supplement: Supplementary file 1 [file jof-11-00225-s001.zip › jof-3527691-supplementary.pdf]

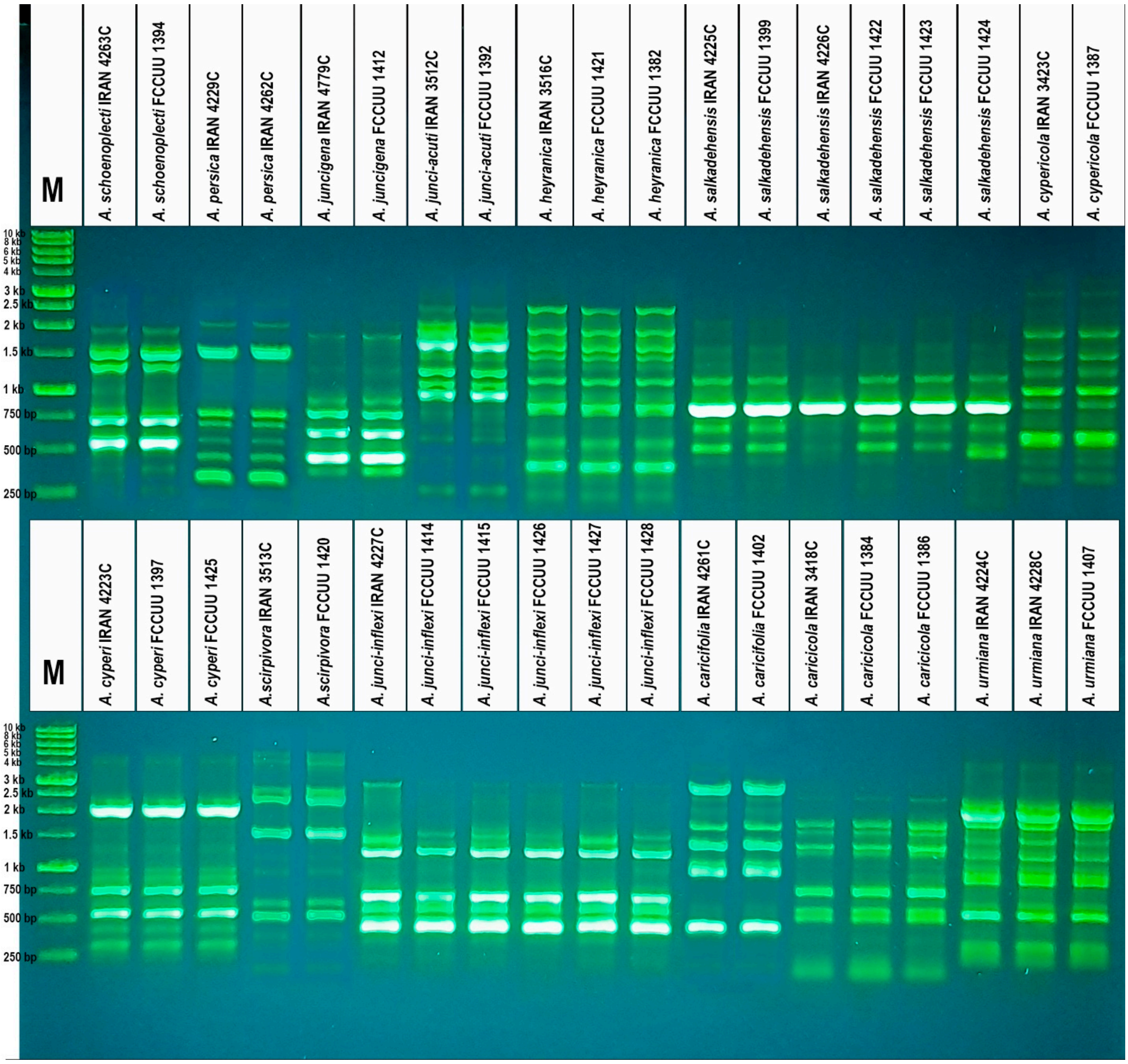


**Supplementary Figure S1.** Banding patterns of different isolates of *Alternaria* section *Nimbya* species using ISSR-PCR with ISSR5 ((GA)$_5$YC) primer. M: 1kb marker
